# Teaching research: a programme to develop research capacity in undergraduate medical students at the University of KwaZulu-Natal, South Africa

**DOI:** 10.1186/s12909-016-0567-7

**Published:** 2016-02-16

**Authors:** Stephen E. Knight, Jacqueline M. Van Wyk, Saajida Mahomed

**Affiliations:** School of Nursing and Public Health, College of Health Sciences, University of KwaZulu-Natal, Room 217 George Campbell Building, Durban, 4041 South Africa; School of Clinical Medicine, University of KwaZulu-Natal, Durban, South Africa; School of Laboratory and Laboratory Medicine, College of Health Sciences, University of KwaZulu-Natal, Durban, South Africa

**Keywords:** Community-based curriculum, Community-oriented primary care, Reflective learning

## Abstract

**Background:**

Improved research ability is a core competency to achieve in health professionals. The Selectives is a three-year, longitudinal, community-based programme within the undergraduate curriculum which aims to develop research capacity in all medical students during the prescribed curriculum. In relation to the programme, the authors describe the types of studies conducted by students, conditions that facilitated their learning, how the experience improved students’ knowledge of research and public health and their development of reflective learning practices.

**Methods:**

A cohort of 212 students completed the Selectives Programme in 2014, and 69 (32 %) completed an anonymous online evaluation thereafter. Data collected include students’ perceptions of the research component of Selectives; its impact on their knowledge of research and a documentary analysis of their research protocols and posters. Ethical approval for the ongoing evaluation of the Selectives was sought and obtained from the institutional Biomedical Research Ethics Committee.

**Results:**

During Selectives, 75 groups of 2–4 students conducted research studies of primary health care problems in community settings. Each group is assessed on their presentation of research findings as a scientific poster. The Selectives facilitated learning for the majority of the cohort. Students reported positive learning experiences about the research process, including ethics; protocol writing; data processing; dissemination of findings and results; and their use in informing a health promotion intervention. Students reported having gained a better understanding of their strengths and weaknesses through reflective learning from this academic activity. The Selectives is scheduled adjacent to the students’ mid-year vacation. This scheduling together with the placement in the students’ home community minimizes travel and accommodation costs associated with working outside the academic teaching platform and therefore makes it a cost-effective model in a low resource context.

**Conclusions:**

The Selectives has proven beneficial to develop a range of generic and practical research competencies for a full cohort of students enrolled in the undergraduate medical curriculum. The Selectives research process is integrated with learning about population health and the social determinants of health in a primary health care setting.

**Electronic supplementary material:**

The online version of this article (doi:10.1186/s12909-016-0567-7) contains supplementary material, which is available to authorized users.

## Background

Aging, a lack of effective training and inadequate exposure to research opportunities for medical students are factors that have contributed to the global and local decline in the number of physician scientists, those who know and apply scientific method to study clinical or health related issues [1–4]. There are furthermore limited resources for the development of these competencies in countries faced with human resource constraints, a huge burden of disease and critical health system challenges [5].

The imperative to improve medical students’ research capacity and reflective learning is one of three educational concerns for medical schools globally [6, 7]. The other priorities are to improve students’ understanding of population health [6, 8] and their knowledge of the ‘upstream’ or social determinants of health and disease [8, 9].

The call to improve ‘scholarship’ in healthcare professionals is one of the attributes being advanced by the Health Professions Council of South Africa (HPCSA) [8]. This attribute encompasses a range of aptitudes linked to the research process, evidence-based practice, health promotion, educating patients and communities, and the development of reflective learning (Table 1).

**Table 1:** Selectives programme research activities aligned to HPCSA scholarship competencies

Scholarship competencies	Research outcomes
• Phrase a clear, answerable, relevant research question related to clinical practice. • Locate, critically evaluate and interpret relevant & previous research findings from robust sources.	Module 1 (2^nd^ year)
	1. Formulate a research question (based on a practice profile & community diagnosis) in a PHC setting
	2. Literature review
	3. Reflective learning
• Consider the applicability of research, understand research design, analysis, and research ethics, consider patient autonomy, respect plagiarism, confidentiality and ownership of intellectual property. • Create, apply, translate and disseminate knowledge.	Module 2 (3^rd^ year)
	1. Prepare a research protocol
	-Online ethics certificate
	-Prepare a questionnaire
	2. Obtain ethical approval from IRB
	3. Conduct fieldwork with informed consent
	4. Process data
	5. Prepare scientific poster
	- Present poster to peers
	6. Reflective learning
• Demonstrate a lifelong commitment to reflective learning.	Module 3 (4^th^ year)
	1. Prepare health promotion intervention informed by research findings
	2. Implement and evaluate the intervention
	3. Reflect on lessons learnt
	4. Orientate incoming 2^nd^ year students to the Selectives research

Early exposure of undergraduate medical students to practical research activities and capacity development have been linked to improved attitudes and scholarship. These students are more likely to pursue research-related careers [10] and become physician scientists, [11] [10] which promotes scientific output [11, 12]. A better understanding of research can help the development of higher order learning such as critical-thinking, problem solving, interpreting data and communicating research findings [10, 13, 14]. For clinicians the benefits of improved research capacity and use of evidence-based approaches for decision making should result in better clinical care, safety and competency [15]. These benefits justify the integration of research teaching into medical curricula [15].

Barriers to research training in undergraduate curricula such as a lack of initiative (exposure, experience and knowledge), impulse (time and competitive environment), incentive (presentation/publication opportunities and acknowledgement) and idols (mentors and supervisors) had been highlighted in the ‘Four I’s’ framework of Scaria [5]. All these factors are very real challenges especially in resource constrained setting when constructing authentic medical curricula which incorporate research training.

A variety of methods have been used to teach research. These include problem-based curricula, research electives, compulsory research projects, and programmes for volunteers and the facilitation of research training by charitable non-governmental organisations. Many initiatives to improve research capacity are ‘add-ons’ for academically talented medical students [16], such as the United Kingdom based extracurricular student-led national collaborative research training initiative [17, 18] or are part of student selected components (SSC’s) of learning [19].

Locating population-based research training within primary health care (PHC) settings advances the social accountability agenda [20]. There are, however, limited descriptions of innovative ways to engage students in ‘real-life’ population-based PHC research and even fewer that link research training to a community-based academic programme [16].

Precedence should thus be given to academic activities that integrate research into the curriculum, incorporate reflective learning, and create an awareness of a population perspective on clinical problems and the influence of the social determinants on health and disease.

A theme advanced by the Medical Education Priority Initiative (MEPI) [21] is the development of research capacity for health care professionals by facilitating locally relevant research training that would augment national research capacity, faculty development and retain researchers in Africa [22–25]. Against this background, the MEPI project at the University of KwaZulu-Natal (UKZN), South Africa [21] has encouraged the development of scholarship and research training for health professionals and specifically medical students [21, 22].

Research teaching and practice at UKZN, has been integrated with PHC clinical teaching in the six year undergraduate medical curriculum. In the next section we provide an overview of the Selectives, with a particular focus on the research process. In the rest of the paper, we present the types of studies conducted by medical students, conditions that facilitated their learning and how the experience improved their knowledge and perceptions of research and public health, and their development of reflective learning practices in the Selectives.

### The selectives programme at UKZN

**Fig. 1:**
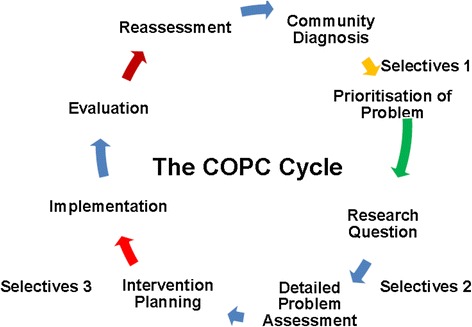
Community-oriented primary care cycle and its relationship to the Selectives Programme

Using the Community-Oriented Primary Care (COPC) approach the Selectives aims to develop medical students who are responsive to the needs of local communities and who will become socially accountable and transformative agents of change in the struggling South African healthcare system (Fig. 1). Moving from the traditional disease focused approach, COPC responds to measured community needs that are addressed through PHC interventions including the ‘upstream’ and social determinants of health [26].

The Selectives comprises three, 4-week modules in the second, third and fourth academic years of the undergraduate medical curriculum (Fig. 1). Successful completion of these modules is a pre-requisite for graduating from the medical programme. Clear learning outcomes are prescribed in the curriculum that include the research process and incorporate ‘scholarship’ competencies (Table 1).

The 4-week period is scheduled to follow the students’ mid-year vacation. This together with the location in the students’ home community minimizes travel and accommodation costs associated with working outside the academic teaching platform and therefore makes it a cost-effective model in a low resource context.

Students choose their own Selectives site, usually near their home town, and work in self-selected groups of two to four. In the first Selectives module they make a community diagnosis [27] based on a ‘practice profile’ [28] of the PHC patient consultations they observe. From this they formulate a relevant PHC research question. Each student then prepares a referenced literature review around the selected research topic. During the second Selectives module in the 3^rd^ academic year each group prepares a research protocol informed by structured lectures and tutorials on basic research methods. A quiz, testing knowledge and application of basic epidemiological concepts including descriptive biostatistics and research methods, is administered before and after a week of lectures to assess students’ learning.

The research protocols are submitted to the institutional ethics review board (IRB) for approval. To facilitate the approval of a large number of undergraduate research studies, the IRB limits the scope of research to questions of a non-sensitive nature and study participants from non-vulnerable populations. With informed consent, data is collected on a custom-designed questionnaire from at least 100 participants in the Selectives Site community. Data from the questionnaire is processed and presented as a scientific research poster to peers and examiners. In the third Selectives module in the 4^th^ year a health promotion activity informed by the research findings is implemented thus closing the research cycle (Fig. 1).

## Methods

An observational descriptive case-study of the Selectives research training for medical students at UKZN was conducted. Some basic demographic variables and the research topics, protocols, and posters produced by this cohort of medical students were analysed. The students, who concluded Selectives in 2014, were reminded on three occasions to complete an anonymous online (Google Forms) custom-developed evaluation questionnaire(Additional File 1). Both quantitative and qualitative data were collected, which enabled students to reflect on their experiences of learning about health research. Questions covered aspects of scholarship and the research process specified in the HPCSA framework. In addition, the questionnaire elicited responses from students about their understanding of research, evidence-based medicine and reflective learning (Table 1). The questionnaire used a 5-point Likert scale to quantify students’ responses. The students’ responses to the stated items were re-categorised as: “Agree”; “Neither agree nor Disagree”; and “Disagree” for ease of reporting. The qualitative data were analysed thematically to determine students’ experience and learning with reference to the HPCSA research framework.

Ethical approval for the ongoing evaluation of the Selectives was sought and obtained from the Biomedical Research Ethics Committee of the UKZN (R201/04).

## Results

The cohort of 212 medical students completed all the requirements of the three Selectives modules in 2014. Most were female (62 %), Black African (70 %) with an average age of 22 years. Students were from areas categorised as rural (37 %), peri-urban or township (27 %) and urban (36 %). The areas where they chose to conduct research reflected the locations of their home categories *i.e*. (39 % rural, 29 % peri-urban and 32 % urban respectively). This is supported by 83 % of the class indicating that they had slept at home during the Selectives period as compared to 29 % who normally sleep at home during term-time.

Each of the students gained in knowledge and application of epidemiological concepts and research methods based on their improved performance in a pre-and post-test (mean: 57 % to 69 % respectively); completed an online ethics certificate; and wrote a literature review. All 75 groups submitted a research study protocol for review and approval, conducted research and presented their results as a scientific poster to peers and faculty members for assessment. When asked about the quality of the protocols, the IRB reviewers commented that many were “better than average.” The scope of topics researched by students reflects the common presenting problems in PHC facilities in KZN (Table 2). Most of the research questions surveyed aspects relating to patients’ knowledge and management of a disease (particularly lifestyle modification), and adherence to medicines.

**Table 2:** Research topics (number) studied by 66 medical student groups in primary health care contexts

Non-communicable Disease (50)	Hypertension (30)
	Diabetes (10)
	Alcohol (3)
	Musculoskeletal (2)
	Epilepsy (2)
	Mental health (2)
	Gastro-intestinal (1)
HIV, AIDS and TB (9)	Tuberculosis (4)
	Sexually transmitted infections (1)
	Safe sex (3)
	Pregnancy (1)
Maternal & Child Health (5)	Expanded Program on Immunisation (2)
	Contraceptives (1)
	Nutrition (1)
	Diarrhoea (1)
Injury & Violence (2)	Gender Based Violence (1)
	Motor vehicle collisions and alcohol use (1)

**Fig. 2:**
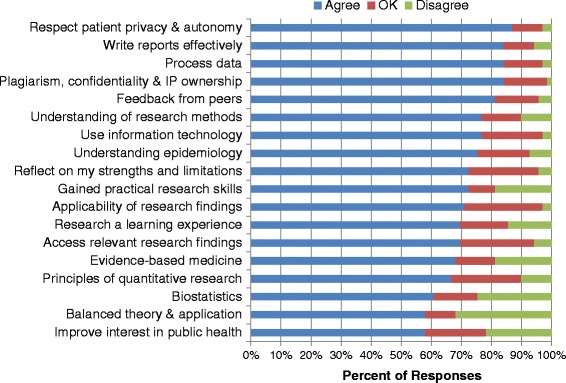
Responses of medical students (*N* = 69) to statements about Selectives research training at UKZN

Sixty nine (32 %) students completed the final Selectives evaluation. The demographic profile of the respondents reflected the diversity of the cohort. The perceptions and experiences of the respondents attest to their increased knowledge and understanding of research (Fig. 2).

The Selectives experience allowed respondents opportunities to learn about research and to gain practical research skills. Students gained a better understanding of both the research methods and process. They improved their understanding of epidemiology and of descriptive biostatistics. Their reported awareness of plagiarism, intellectual property, ethics and study participant confidentiality had increased. These students also developed skills in accessing and appraising literature relevant to their research topics; processing and analysing data; and writing and presenting evidence-based reports. From the health promotion interventions conducted, they appreciated the applicability of research findings. Qualitative comments attest to students’ learning in the five Scholarship competencies (Table 3).

**Table 3:** Students’ comments of the selectives programme development of their scholarship competencies

Scholarship competency	Students’ qualitative comments
Phrasing clear, answerable, relevant research questions related to clinical practice.	• *[The Selective helped me in…] identifying common problems and doing research on this in the community*
Locating, critically evaluating and interpreting relevant & previous research findings from robust sources	• *Learning how to [conduct] and write a literature review*
Considering the applicability of research, understanding research design, analysis, research ethics, consider patient autonomy, respect plagiarism, confidentiality and ownership of intellectual property.	• *The Selectives has given us abilities and skills in research that we would not have gained anywhere else in our medical curriculum and even some qualified doctors don’t have and are shocked that we have such a good approach to research* • *Health promotion based on what we found in the research*
Creating, applying; translating and disseminating knowledge.	• *Able to apply the knowledge that I have learnt on the classroom in a real clinical setting* • *I improved my presentation skills* • *It was great publishing our research in a journal*
Demonstrating a lifelong commitment to reflective learning.	• *I felt like we made a difference during the health promotion intervention.* • *It helped me to reflect on myself as a clinician, I liked the use of the Gibbs [reflective learning] Cycle.* • *Valued the constructive feedback from peers and lecturers on our health promotion intervention*

During the Selectives modules the students also learned about reflective practice. They valued the feedback received from their peers and perceived the group process as supportive of their learning. The majority of the respondents reported that the Selectives had offered them sufficient opportunities to learn and practice self-reflection.

Most students valued the learning platform provided by the community-based sites as it assisted them in measuring the burden of disease; applying clinical epidemiology while gathering authentic patient information; developing knowledge and skills to evaluate the PHC services; and in understanding the role of the practitioners who rendered services on the platform. The Selectives further developed generic skills including time management; teamwork, self-directed learning and the use and application of information technology which is essential to successful research.

The best research posters and presentations are awarded prizes. A number of groups have presented their research at National and local College Research meetings. Two groups have prepared *Scientific Letters* to local journals and most groups gathered data of sufficient quality to do this with appropriate motivation and support [29]. Students are encouraged to display their research posters at clinics in the Selectives sites.

## Discussion

During Selectives students conduct authentic research in community-based and decentralized learning settings. A unique feature of this programme is the location of the research setting i.e. within the home community of the student. The community-based placement and timing of the Selectives facilitates the teaching and supervision by a multi-professional team of teachers outside the faculty addressing the recommendation of the Frenk report, which requires health professions education to be more PHC focused [6]. In line with the goal to encourage and advance health professional education and training in decentralized, rural training sites, we believe the success of the Selectives lies in the fact that two thirds of students conducted research in sites situated in rural or disadvantaged communities [30].

In this programme research capacity development is integrated into the curriculum and the completion of the research modules is a prerequisite for graduation. The importance and mandatory inclusion of research training in undergraduate curricula has been highlighted by the Boyer Commission on Educating Undergraduates in the Research University [31, 32]. In Germany the completion of a dissertation is required before students can be awarded their medical degree [31]. Only a small proportion of these German medical students successfully complete the thesis during their clinical training and it was proposed that basic training in scientific research methodology should become an integrated part of their medical curriculum [33].

Students recognise the benefits of research experience [19, 34, 35]. In the Selectives, students highly valued the research experience for exposing them to translational research in a manner that is inseparable from patient contact and clinical care. The experience is believed to be efficient as it also informed subsequent health promotion interventions planned by the students. The Selective thus provides an authentic and useful research experience to respond to a challenge to include research training in the design of the modern medical curriculum.

Each group presented their research in a setting that simulated the process following the acceptance of an abstract by a peer-reviewed scientific conference. Poster sessions are time tabled and students present to faculty, site supervisors and peers. For some, the quality of the research has also been of sufficient standard for dissemination as indicated by those accepted for publication in peer reviewed journals [29]. This finding probably attests to the skills of their faculty supervisors and their guidance to the completion of the research cycle. The quality of the UG student research is different to a survey conducted at the University of Cape Town, where staff perceived student research as substandard and not worthy of presentation [3].

However, there are challenges in securing adequate numbers of suitably trained faculty supervisors to guide the groups. Due to the bulky nature of medical curricula and the off-site location of the implementation of the research, groups and supervisors complain of time pressures to prepare the research protocols and posters within short deadlines. The majority of students accepted on the medical programme are first time tertiary education entrants with limited prior research exposure and many are English second language users. For some these barriers impact on the quality of their research outputs.

The students in our study completed ‘real research’ and perceived themselves as more experienced in information retrieval and writing skills. This finding resonates with others who compared the perceptions of students in research tracks to those who participated in basic skills electives or lecture-based courses [36]. Previous studies also suggests that medical students with research experience in primary care settings are more likely to pursue research related careers and thus more uniquely placed to conduct population-based studies [10]. Yet, in the absence of literature describing innovative ways to engage students in population-based primary care research, it is hoped that this study will encourage health professions educators in similar resource constrained settings.

The Selectives emphasises active and student-centred learning and equips students with skills for evidence-based research and practice of EBM. By addressing the barriers as highlighted in the ‘Four I’s’ framework, [5] the Selectives programme successfully addresses each of the barriers and promotes social accountability that facilitates students’ transition to becoming transformative learners.

Although the proportion who completed the end of programme questionnaire was low, the demographic profile of the respondents reflected that of the cohort. The level of response is also in keeping with the average rate reported for “end of module” evaluations on the undergraduate programme.

The sustainability of the Selective relates to the feasible costing, incremental and longitudinal research exposure for every undergraduate student in a primary health setting that is curriculated.

## Conclusions

The Selectives Programme is an effective way to develop and incorporate research training; at minimal cost to the faculty, for undergraduate medical students in community-based (de-centralized) PHC learning platforms. This community engagement early in the curriculum extends the learning platform, exposes students to a variety of PHC facilitators and learners play a more active role in their learning. The Selectives enabled students to integrate their community and population perspective and clinical training using the COPC cycle. The longitudinal acquisition of knowledge and skills through researching authentic PHC problems in their ‘home’ communities has been beneficial to students’ understanding of local needs and exposes them to the advocacy role as possible change agents for the community. Students use the findings of their research to implement appropriate health promotion interventions according to identified needs.

The Selectives Programme has evolved into the current format over the last decade and there is a need for a more systematic process to benchmark students’ gain in research experience. Further research with communities and healthcare workers at clinics will help us understand the extent to which research and interventions by undergraduate students are beneficial to the PHC providers and communities. Graduate tracking is essential to assess the number of health care workers who become physician-scientists as a result of being taught research as undergraduate medical students.

### Ethical approval

Ethical approval for the study was sought and obtained from the Biomedical Research Ethics Committee of the UKZN (R201/04).

## Additional file

Additional file 1:
**Questionnaire.** (PDF 110 kb)
