# Supplementary material for: Teaching research: a programme to develop research capacity in undergraduate medical students at the University of KwaZulu-Natal, South Africa
Source: BMC Med Educ. 2016 Feb 16;16:61. doi: 10.1186/s12909-016-0567-7 (PMC4754994; doi:10.1186/s12909-016-0567-7)
Supplement: Additional file 1: — Questionnaire. (PDF 110 kb) [file 12909_2016_567_MOESM1_ESM.pdf]

Edit this form

EVALUATION OF SELECTIVES PROGRAMME September 2015

Congratulations on having completed Selectives 01, 02 AND 03.
In this anonymous Google Forms questionnaire you will be asked your opinion on various aspects of the Selectives Programme for the purposes of evaluation. Student Evaluation is one of the methods used for improving the quality of teaching at the university. This survey will provide your lecturers with valuable feedback about teaching effectiveness.
This is an ANONYMOUS questionnaire - Please complete as accurately and honestly as possible.
It should take you about 20 to 30 minutes to complete comprehensively
Thank you for your co-operation.

In each question you are presented with a statement to which you should indicate the EXTENT of your agreement/disagreement. Please respond appropriately and honestly
THIS IS YOUR OPPORTUNITY TO GIVE US CRITICAL AND CONSTRUCTIVE CRITICISM
Thank you in anticipation
Selectives Team

* Required

Gender *

Are you:

Age *

Are you

Selectives Site *

Is your Selective Site based in a:

Selectives Programme Evaluation *

What do you think of the Selectives Programme?

	No I strongly dIsagree	Disagree	Agree	Yes, I strongly agree
I thought the Selectives Programme was well organized.	○	○	○	○
The Moodle assignment instructions and Student Manual provided detailed information on what was required of me in Selectives	○	○	○	○
I understand how the 3 Selectives modules fit together into the Community-oriented Primary Care (COPC) approach	○	○	○	○

	No I strongly dIsagree	Disagree	Agree	Yes, I strongly agree
Selectives has a good balance between the theory and its application during the 3- Selectives Blocks	○	○	○	○
The assignments helped me learn about all aspects of the Community-oriented Primary Care (COPC) approach	○	○	○	○
I understand what is meant by a population perspective on health	○	○	○	○
I appreciated the integration of clinical primary care and a population perspective	○	○	○	○
The workload was manageable.	○	○	○	○
The Selective Site teaching platform is conducive to learning.	○	○	○	○

University Selectives Supervisor Evaluation *

What do you think of your Selectives Supervisor?

	No I strongly dIsagree	Disagree	Agree	Yes, I strongly agree
The Selectives Supervisor was sensitive to the different cultural backgrounds of the students.	○	○	○	○
My Selectives Supervisor was approachable	○	○	○	○
My Selective Supervisor was available .	○	○	○	○
I received sufficient guidance to cope with the Selectives modules	○	○	○	○

Selectives Lecture Evaluation *

What do you think of the Selectives Lectures?

	No I strongly dIsagree	Disagree	Agree	Yes, I strongly agree
The lectures were clear and well-explained	○	○	○	○
The lectures stimulated my enthusiasm for public health.	○	○	○	○

Selectives Site Facilitator Evaluation *

What do you think of the Selectives Site Facilitator?

	No I strongly dIsagree	Disagree	Agree	Yes, I strongly agree
My Selectives Site Facilitator was approachable.	○	○	○	○
My Selectives Site Facilitator was available	○	○	○	○
I learnt about a lot from my Selectives Site Facilitator	○	○	○	○

Selectives Assessment Evaluation *

What do you think of the Selectives Assessment?

	No I strongly dIsagree	Disagree	Agree	Yes, I strongly agree
The assessment criteria were clear from the start.	○	○	○	○
There is a good link between what we learned in the module and how we were assessed.	○	○	○	○
The time and effort required to complete the assessment tasks was reasonable.	○	○	○	○
Useful feedback was provided on assignments.	○	○	○	○

Selectives Site Evaluation *

What do you think of the Selectives Site?

	No I strongly dIsagree	Disagree	Agree	Yes, I strongly agree
I have gained practical public health skills at my selective sites.	○	○	○	○

PUBLIC HEALTH AND RESEARCH TEACHING *

What do you think of the Research acivitiy?

	Yes, greatly	yes	No	No, not at all
Was the Selectives Research Study a useful learning experience?	○	○	○	○
Did the Selectives improve your understanding of research methods?	○	○	○	○
Did the Selectives Programme improve your understanding of epidemiology?	○	○	○	○
Did the Selectives Programme improve your understanding of the social ('upstream') determinants of health?	○	○	○	○
Did the Selectives Programme improve your interest in public health?	○	○	○	○
Did the Selectives Programme improve your interest in health research?	○	○	○	○
Did the Selectives Programme stimulate you to engage with community health issues with friends or family outside of class?	○	○	○	○
Did the Selectives Programme challenge your thinking about medicine?	○	○	○	○
Has the Selectives Programme made you more aware and concerned about societal problems?	○	○	○	○

Selectives and Core Competencies of a Health Professional *

The Selectives Programme has enabled me to:

	No I strongly dIsagree	Disagree	Agree	Yes, I strongly agree
Perform a primary health care clinical consultation effectively	○	○	○	○
Work in primary healthcare settings	○	○	○	○
	○	○	○	○

	No I strongly dIsagree	Disagree	Agree	Yes, I strongly agree
Understand the environmental factors that influence health and disease in primary health care settings.				
Understand the patinets context better	○	○	○	○
Include prevention and health promotion in the patients management plan.	○	○	○	○
Have a working understanding of Standard Treatment Guidelines and Essential Drug List protocols.	○	○	○	○
Understand the structure, organisation and function of the South African healthcare system	○	○	○	○

Selectives and Communication *

The Selectives Programme has enabled me to:

	No I strongly dIsagree	Disagree	Agree	Yes, I strongly agree
Understand a patient-and community-centred approach to interactions with patients and their families.	○	○	○	○
Respect patient confidentiality, privacy and autonomy.	○	○	○	○
Motivate patients and their families and communities to take personal responsibility for their health.	○	○	○	○
Gather information about health conditions, as well as about a patient's beliefs, concerns, expectations and illness experience.	○	○	○	○
Collect and process data	○	○	○	○
Reflect on the importance of respect for diversity and	○	○	○	○

	No I strongly dIsagree	Disagree	Agree	Yes, I strongly agree
difference, including but not limited to the impact of ethnicity, gender, religion, education and culture in medical practice.				
Present written reports of clinical encounters and plans effectively, using language, visual, information technology and numeracy skills.	○	○	○	○

Selectives and Teamwork *

The Selectives Programme has enabled me to:

	No I strongly dIsagree	Disagree	Agree	Yes, I strongly agree
Appreciate professional diversity and demonstrate the ability to work with the others in the healthcare team.	○	○	○	○
Work interdependently in my group and share tasks with others to assess, plan, provide and integrate quality care for individual patients or groups of patients.	○	○	○	○
Have an understanding of home and community-based care in, with insight into the potential contributions of community support groups.	○	○	○	○
Evaluate the burden of disease within the community using local data.	○	○	○	○
Evaluate existing primary healthcare services and practitioners.	○	○	○	○
Collaborate with other professionals, relevant organisations and the community to draw up a plan to manage the	○	○	○	○

	No I strongly dIsagree	Disagree	Agree	Yes, I strongly agree
identified health priorities and collectively promote health.				

Selectives and being a Scholar *

The Selectives Programme has enabled me to:

	No I strongly dIsagree	Disagree	Agree	Yes, I strongly agree
Reflect on, and acknowledge, the strengths and limitations of my knowledge and skills.	○	○	○	○
Be able to write comprehensive, complete and accessible records for the purposes of good clinical practice.	○	○	○	○
Effectively and efficiently access relevant research findings from robust sources.	○	○	○	○
Consider the applicability of research findings to a particular context.	○	○	○	○
Understand the basic principles of quantitative research design and analysis, and research ethics.	○	○	○	○
Respect and comply with laws pertaining to plagiarism, confidentiality and ownership of intellectual property when accessing and using information and when conducting research.	○	○	○	○

Selectives and being a Leader and Professional *

The Selectives Programme has enabled me to:

	No I strongly dIsagree	Disagree	Agree	Yes, I strongly agree
Respect and comply with laws pertaining to plagiarism, confidentiality and ownership of	○	○	○	○

	No I strongly dIsagree	Disagree	Agree	Yes, I strongly agree
intellectual property when accessing and using information and when conducting research.				
Select effective teaching strategies and content to facilitate patient and community learning.	○	○	○	○
Reflect on teaching / presentation encounters and use feedback from peers and others to guide my development as an effective communicator.	○	○	○	○
Create an enabling and supportive learning environment that is sensitive to issues that can influence learning in communities.	○	○	○	○
Maintain professional competence through on-going self-reflection and peer review.	○	○	○	○
Understand appropriate professional, legal and ethical codes of practice of health care professionals.	○	○	○	○

Selectives and being a Health Advocate *

The Selectives Programme has enabled me to:

	No I strongly dIsagree	Disagree	Agree	Yes, I strongly agree
Identify the health needs of a community taking into consideration the context of the community.	○	○	○	○
Act as an advocate for patient groups with particular health needs (including the poor and marginalised	○	○	○	○

	No I strongly dIsagree	Disagree	Agree	Yes, I strongly agree
members of society).				
Familiarise myself with the communities we serve by obtaining insight into local cultures and belief systems as these relate to the understanding of health and disease	○	○	○	○
Identify opportunities for, health promotion and disease prevention within the context of promoting a healthy environment and lifestyle.	○	○	○	○

Selectives and Public Health *

The Selectives Programme has given me an: A.; Inadequate B. Appropriate; OR C. Excessive; understanding of:

	Inadequate	Appropriate	Excessive
Public health	○	○	○
Role of social service agencies in PHC	○	○	○
Infectious disease prevention including immunization	○	○	○
Biostatistics.	○	○	○
Evidence-based medicine.	○	○	○
Environmental health.	○	○	○
Social determinants of health	○	○	○
Primary Health Care policy.	○	○	○
Health care system.	○	○	○
Behavioural sciences.	○	○	○

Comments

What THREE (3) aspects of the Selectives Programme did you really like or find most useful?

Comments

What THREE (3) things about the Selectives Programme did you not like or found least useful?

Comments

What THREE (3) suggestions do you have as to how the Selectives Programme can be improved?

Submit

Never submit passwords through Google Forms.

100%: You made it.

Powered by

This content is neither created nor endorsed by Google.

Report Abuse - Terms of Service - Additional Terms
